# Metabolic Syndrome and Stroke Recurrence in Chinese Ischemic Stroke Patients – The ACROSS-China Study

**DOI:** 10.1371/journal.pone.0051406

**Published:** 2012-12-05

**Authors:** Donghua Mi, Qian Jia, Huaguang Zheng, Kolin Hoff, Xingquan Zhao, Chunxue Wang, Gaifen Liu, Yilong Wang, Liping Liu, Xianwei Wang, Yongjun Wang, On behalf of the investigators for the survey on Abnormal Glucose Regulation in Patients with Acute Stroke Across China (ACROSS-China)

**Affiliations:** 1 Department of Neurology, Beijing Tiantan Hospital, Capital Medical University, Beijing, People’s Republic of China; 2 Division of Endocrinology, Diabetes and Metabolism Perelman School of Medicine, The University of Pennsylvania, Philadelphia, Pennsylvania, United States of America; University of Münster, Germany

## Abstract

**Objective:**

Metabolic syndrome has emerged as a novel risk factor in cardiovascular disease due to its potential for predicting stroke in population-based studies. We investigated the relationship of metabolic syndrome with stroke recurrence.

**Methods:**

This was a retrospective analysis of Chinese patients enrolled in the prospective Abnormal gluCose Regulation in patients with acute strOke acroSS China (ACROSS-China) study after their first ischemic stroke. Metabolic syndrome was defined using the International Diabetes Federation (IDF) criteria. Vascular risk factors were assessed. Outcome was defined as recurrence of stroke within one year after the index ischemic stroke. Cox proportional hazards regression was performed to identify potential predictors of stroke recurrence.

**Results:**

The prevalence of metabolic syndrome among 2639 ischemic stroke patients was 51.35%. During the one-year follow-up, 195 strokes (7.4%) recurred. The multivariate hazard ratio (95% CI) of stroke recurrence was 1.94 (1.39–2.73) for metabolic syndrome. After adjustment for components, metabolic syndrome lost its association with stroke recurrence; in this model, high fasting plasma glucose (IDF definition) was a predictor for stroke recurrence.

**Conclusion:**

Metabolic syndrome may not be predictive for stroke recurrence beyond its component individual factors for Chinese ischemic stroke patients.

## Introduction

Despite improved treatments for managing vascular disease risk factors, recurrent vascular events still occur in a high proportion of patients following strokes. Several studies have assessed the risk of stroke recurrence after a vascular event. Data from the CAPRIE study (Clopidogrel versus Aspirin in Patient Risk of Ischaemic Events, CAPRIE) [1] and REACH study (The REduction of Atherothrombosis for Continued Health, REACH) [2] showed that the stroke recurrence rate is about 6% annually. Data from the Chinese National Stroke Registry found that the stroke recurrence rate in patients with ischemic stroke was 16% in the first year, combined with a vascular event recurrence rate of 18% [3].

Previous studies have divided risk factors for stroke recurrence into three categories: the first category is comprised of immutable risk factors [4], including age, gender, race, and heredity; the second category includes risk factors that can be intervened, such as hypertension [5], [6], diabetes mellitus [7], smoking [8], and atrial fibrillation [5]; the third category consists of newly discovered risk factors that differ from traditional risk factors, and include elevated homocysteine [9], hypercoagulable states [10], patent foramen ovale [11], and metabolic syndrome [12].

Metabolic syndrome has insulin resistance as the core mechanism and abdominal obesity as the prominent clinical manifestation. The worldwide prevalence of metabolic syndrome is high and increasing. Approximately one quarter of adults in developed countries have metabolic syndrome [13], [14]. Approximately 11% of Chinese adults currently have metabolic syndrome, although as China develops economically, the prevalence of metabolic syndrome is predicted to increase [15]. Guidelines for ischemic stroke and transient ischemic attack (TIA) published in 2006 [16] and 2011 [17] by the American Heart Association and American Stroke Association indicate that metabolic syndrome can predict coronary heart disease, cardiovascular disease (including coronary heart disease and stroke), and resulting mortality. However, only a few studies have reported on the relationship between metabolic syndrome and stroke recurrence risk [18], [19]. The potential significance of metabolic syndrome in predicting recurrent cerebrovascular events is not well documented and we believe that further research is needed to clarify this association. The objective of the present study was to address metabolic syndrome in Chinese patients with ischemic stroke.

**Table 1:** Subject baseline characteristics and one-year stroke recurrence according to the presence of metabolic syndrome^a^.

	Metabolic syndrome (n = 1355)	No metabolic syndrome (n = 1284)	*P* value^b^
Female (%)	44.2	27.3	0.00
Median Age (interquartile range), years	62 (53–72)	64 (54–74)	0.01
Smoker (current or former) (%)	39.5	47.3	0.00
History of atrial fibrillation (%)	5.3	7.8	0.01
History of coronary heart disease (%)	15.1	12.5	0.05
Median NIHSS^h^ at admission (interquartile range)	4 (2–8)	4 (2–8)	0.65
Elevated waist circumference^d^ (%)	73.2	23.4	0.00
Elevated TG^e^ (%)	60.8	13.9	0.00
Low HDL-C^f^ (%)	65.6	22.8	0.00
Elevated BP^c^ (%)	87.5	60.4	0.00
Elevated fasting blood glucose^g^ (%)	73.8	27.1	0.00
Stroke recurrence	21.5	12.4	0.00

BP, blood pressure; TG, triglyceride; HDL-C, high-density lipoprotein cholesterol; NIHSS. National Institutes of Health Stroke Scale.

a Metabolic syndrome was defined by the International Diabetes Federation (IDF) criteria.

b Wilcoxon rank sum tests and Pearson Chi square tests used for continuous and categorical variables, respectively.

c Systolic blood pressure ≥130 mmHg or diastolic blood pressure ≥85 mmHg, or medication.

d Waist circumference ≥80 cm for women or ≥90 cm for men.

e Triglyceride level ≥150 mg/dL, or medication.

f HDL-C level ≤50 mg/dL for women or ≤40 mg/dL for men, or medication.

g Fasting glucose level ≥100 mg/dL or medication for elevated glucose or antidiabetic medication.

h Neurologic impairment.

## Methods

### Study population

Patients enrolled in the Abnormal gluCose Regulation in patients with acute strOke acroSS China (ACROSS-China) trial were retrospectively evaluated for this study. The ACROSS-China study was a prospective, multicenter cohort investigation designed to determine the prevalence of impaired glucose tolerance (diabetes) on Day 14 after the occurrence of a patient’s first stroke. Inclusion criteria for ACROSS-China trial were shown as follows: adults of either sex; acute stroke diagnosed using World Health Organization (WHO) criteria; patient within 14 days of experiencing stroke. no previous medical history of stroke. The exclusion criteria were as follows: cerebral infarction without symptoms and signs; non-cerebrovascular causes for the neurologic deficit(s) (primary or metastatic neoplasm, post-seizure paralysis, head trauma, etc.); stroke occurring more than 14 days previously; and refusal of consent by the patient or his/her legal agent.

Patients were recruited consecutively in our study according to inclusion and exclusion criteria for ACROSS-China trial and the following criteria: no acute recurrence (within 14 days) of ischemic stroke; and no intracranial or subarachnoid hemorrhage. Each patient recruited had focal or more general involvement of the nervous system, with associated neurologic deficits.

This ACROSS study included 3450 patients with acute stroke hospitalized at 35 participating centers across China between 2008 and 2009. Details of the ACROSS-China study design and baseline results have been previously published [20]. We excluded 811 participants who had hemorrhagic stroke and acute recurrence of ischemic stroke within 14 days, so that a total of 2639 participants comprised our main study sample, according to our inclusion and exclusion criteria.

The ethics committees of Beijing Tiantan Hospital at all participating centers approved the procedures, and all patients or their designated relatives gave written informed consent.

**Table 2:** Hazard ratios (95% CIs) showing the relationship of metabolic syndrome and their individual components with stroke recurrence^a^.

	NO (%)	Stroke recurrence	
		Multivariate Adjusted HR	*P* Value
Metabolic syndrome	1355 (51.4)	1.94 (1.39–2.73)	0.00
Elevated waist circumference	1293 (49.0)	1.36 (0.97–1.90)	0.07
Elevated TG	1003 (38.0)	1.05 (0.76–1.46)	0.77
Low HDL-C	1182 (44.8)	1.08 (0.78–1.49)	0.64
Elevated BP	1961 (74.3)	0.88 (0.60–1.28)	0.50
Elevated fasting blood glucose	1349 (51.1)	3.71 (2.57–5.37)	0.00

BP, blood pressure; TG, triglyceride; HDL-C, high-density lipoprotein cholesterol; HR, hazard ratios; NO, number.

Multivariable models were adjusted for age, sex, smoking behavior, history of atrial fibrillation and coronary heart disease, NIHSS at admission and medication with antihypertensive and hypoglycemic drugs in hospital.

a Multivariate Cox regression analysis of stroke outcome in patients with ischemic stroke.

### Assessments

Demographics and classical vascular risk factors were recorded, as well as clinical characteristics. The severity of neurologic impairment was evaluated within 24 hours of admission using the National Institutes of Health Stroke Scale (NIHSS) [21]. After the index event, secondary preventive treatments were administered, which included antiplatelet, anticoagulant, antihypertensive, and lipid-lowering therapy.

Patients were contacted by telephone for follow-up interviews by trained research personnel at Beijing Tiantan Hospital. A standardized script was used to collect study data 3 and 12 months after disease onset. When it was impossible to speak to the patient or when the information provided by the patient was deemed unreliable by the interviewer, the caregiver was contacted and interviewed.

We evaluated the association of metabolic syndrome with recurrent stroke during a one-year follow-up period. Stroke recurrence was defined as a new neurologic deficit, including ischemic (or transient ischemic attack) or hemorrhagic stroke, associated with re-hospitalization. If the patients were followed up for stroke recurrence, the sub-centers conducted face-to-face follow-ups and hospital certificates were faxed to the Tiantan Hospital. In cases of suspected recurrent cerebrovascular event without hospitalization, a judgment was made by the research coordinators together with the principal investigator (YW).

### Definition of metabolic syndrome

Metabolic syndrome was defined using International Diabetes Federation (IDF) criteria [22]. Individuals were considered to have metabolic syndrome if they had central obesity (waist circumference ≥90 cm for Asian men or ≥80 cm for Asian women) plus any two of four additional factors. These four factors are:

- Elevated triglyceride (TG) levels: ≥1.7 mmol/l (150 mg/dl);
- Decreased HDL-cholesterol levels: <1.03 mmol/l (40 mg/dl) in males and <1.29 mmol/l (50 mg/dl) in females (or specific treatment for these lipid abnormalities);
- Elevated blood pressure (systolic BP ≥130 or diastolic BP ≥85 mmHg) (or treatment of previously diagnosed hypertension);
- Elevated fasting plasma glucose (FPG) levels [FPG ≥5.6 mmol/l (100 mg/dl)] (or previously diagnosed type 2 diabetes).

Waist circumference was measured between the lower rib margin and the iliac crest after a normal expiratory breath. Triglyceride, HDL-C, and fasting glucose levels were measured at admission. Hyperglycemia and hypertension were determined on the basis of measurements taken on Day 14±3 after the occurrence of stroke. Fasting glucose levels were obtained to avoid stress-related hyperglycemia, which often accompanies the acute phase of stroke. Likewise, blood pressure measurements (conducted while sitting) avoided measurement of stress-related hypertension.

### Statistical analysis

Statistical analyses were carried out with SAS software, version 9.1.3 (SAS Institute, Inc., Cary, NC, USA). Chi square or Wilcoxon rank sum tests were used to determine differences in clinical characteristics among patients with and without metabolic syndrome.

Stroke recurrence rate for stroke survivors was calculated for the 12 months following from the date of stroke onset. Patients were censored at the date of recurrence. The independent contribution of each risk factor to ischemic stroke outcomes was estimated by Cox’s proportional hazards regression model. Clinical covariates with a univariate probability value of 0.05 were entered into the Cox’s proportional hazards regression model to adjust for potential confounders. Values of *P*<0.05 were considered statistically significant.

## Results

### Baseline characteristics and one-year outcomes

Risk factors for the 2639 patients in the study included elevated waist circumference (49.0%), elevated triglycerides (38.0%), low HDL-C (44.8%), high blood pressure (74.3%), and elevated fasting blood glucose (51.1%).

Baseline characteristics for the study participants with or without metabolic syndrome are summarized in Table 1. Metabolic syndrome was present in 51.4% of the participants. Patients with metabolic syndrome were more likely to be female, slightly younger, nonsmokers, and less likely to have a previous history of atrial fibrillation, than subjects without metabolic syndrome (*P*<0.05). Neurologic impairment (NIHSS scores) was not different between subjects with metabolic syndrome and those without. An equal possibility was observed in these two groups of having a previous history of coronary heart disease. At the one-year follow-up, a total of 21.5% of subjects with metabolic syndrome had stroke recurrence compared to 12.4% of those without metabolic syndrome (Table 1). This difference was significant (*P*<0.05) when none of the adjustment factors were taken into consideration.

### Metabolic syndrome’s association with stroke recurrence

According to the analysis in Table 2, the presence of metabolic syndrome was significantly associated with increased risk of stroke recurrence. The adjusted hazard ratio (HR) was 1.94 with a 95% confidence interval (CI) of 1.39–2.73. Further multivariate analyses investigated hazard ratios for stroke recurrence for each of five potential risk factors: elevated waist circumference, increased triglycerides, high blood pressure, elevated fasting glucose, and low HDL-C. The model was adjusted for age, sex, smoking behavior, history of atrial fibrillation and coronary heart disease, and NIHSS at admission. Of the five potential risk factors, only elevated fasting glucose (HR: 3.71; 95%CI: 2.57–5.37) was correlated with risk of stroke recurrence. (*P*<0.05, Table 2).

As shown in Table 3, after adjustment for all metabolic syndrome components, the HR for stroke recurrence became non-significant for metabolic syndrome (Table 3). However, in this model, high fasting plasma glucose (IDF definition) remained the significant predictor for stroke recurrence.

**Table 3:** Hazard ratios of stroke recurrence for metabolic syndrome and individual components in the model, adjusted for metabolic syndrome and its individual components^a^.

	Stroke recurrence	
	Multivariate Adjusted HR	*P* Value
Metabolic syndrome	1.46 (0.69–3.10)	0.33
Elevated waist circumference	0.99 (0.48–2.05)	0.98
Elevated TG	1.01 (0.72–1.41)	0.96
Low HDL-C	1.04 (0.75–1.44)	0.83
Elevated BP	0.83 (0.57–1.23)	0.36
Elevated fasting blood glucose	3.46 (0.69–3.10)	0.00

BP, blood pressure; TG, triglyceride; HDL-C, high-density lipoprotein cholesterol; HR, hazard ratios; NO, number.

Multivariable models were adjusted for age, sex, smoking behavior, history of atrial fibrillation and coronary heart disease, NIHSS at admission and medication with antihypertensive and hypoglycemic drugs in hospital.

a Multivariate Cox regression analysis of stroke outcome in patients with ischemic stroke.

## Discussion

Stroke is characterized by high rates of recurrence and mortality [23], [24]. A growing body of evidence supports metabolic syndrome as a risk factor for a recurrent stroke or future cerebral ischemic event [25]–[28]. However, most research on metabolic syndrome and cerebrovascular disease has been restricted to stroke prevention rather than prognosis. The present study is the first (retrospective) analysis of a relatively large, multicenter, prospective cohort study to consider the impact of metabolic syndrome on the prognosis for ischemic stroke in China.

We demonstrated that metabolic syndrome was associated with an increased risk of stroke recurrence. After further controlling for its components, metabolic syndrome lost its association with stroke recurrence. However, high fasting plasma glucose remained as an independent predictor for stroke recurrence.

The causes of stroke recurrence are multifactorial. Several authors have investigated the effects of potentially modifiable risk factors on stroke recurrence as a means of targeting secondary preventive interventions. The most widely established risk factors associated with stroke recurrence are diabetes mellitus [29] and atrial fibrillation [30]. Epidemiologic studies have shown that metabolic syndrome significantly increases the risk of stroke events, as well as all-cause mortality [31]–[33]. In addition, Arenillas and colleagues reported that metabolic syndrome is associated with resistance to clot lysis after tissue plasminogen activator therapy, and stated that the presence of metabolic syndrome can hamper the arterial recanalization process [34], which is suggestive of a poor stroke prognosis.

The results from our study, showing a prevalence of metabolic syndrome in our cohort of approximately 50% and a hazard ratio for stroke recurrence (metabolic syndrome *vs.* no metabolic syndrome) approaching 2, are generally consistent with those of previous studies. For example, Boden-Albala *et al.* [25], Ovbiagele *et al.* [19] and Liu *et al.* [35] reported values for prevalence of 44%, 43% and 40%, although lower values (14–26%) have also been reported in some studies [18], [27], [28]; this variation may reflect differences in the cohort enrolled, including ethnic origin and the presence or absence of cardiovascular or cerebrovascular disease (including history of previous stroke) at the time of recruitment. Greater consistency is observed with respect to the increased risk of first or recurrent stroke reported in patients with metabolic syndrome (hazard ratios of 1.5–2.4) [19], [25]–[28], [35].

However, there remains some debate as to whether the use of metabolic syndrome as a predictor of stroke (either first or recurrent) offers any advantages over the use of the individual components that underlie its definition. Ovbiagele *et al.* found that controlling for metabolic syndrome’s individual factors did not reveal a significant relationship for stroke or combined outcomes for patients with intracranial stenosis [19]. Similarly, Callahan *et al.* did not observe a significant increase in the risk of stroke or major cardiovascular event in patients with metabolic syndrome who had experienced a previous stroke or transient ischemic attack [18]. Liu and colleagues reported that, of the component factors of metabolic syndrome, hypertension, hyperglycemia and impaired fasting glucose were independent risk factors for cardiovascular disease in patients with a previous ischemic stroke [35], consistent with the findings of Koren-Morag and coworkers that impaired fasting glucose and hypertension were the strongest predictors of first stroke or transient ischemic attack [27]. In our study, metabolic syndrome was not associated with an increased risk of stroke recurrence after controlling for its components, although elevated fasting plasma glucose remained a robust predictor. This is consistent with the results of epidemiologic studies that have evaluated the relationship between diabetes and stroke recurrence [31]–[33]. In the China National Stroke Registry, patients with diabetes had a significantly higher incidence of recurrent stroke at 3 and 6 months after stroke onset [36].

Although obesity, defined on the basis of either waist circumference (as was done in this study) or body mass index, is a fundamental component of metabolic syndrome, it is interesting that certain studies have reported that obesity may be a protective factor, in patients experiencing a first cardiovascular event, against recurrence of a cardiovascular event (including stroke), a phenomenon known as the ‘obesity paradox’ [37], [38]. However, it has been argued that this paradox may, in fact, reflect either the limitations of the methods used to define obesity, or the introduction of bias due to the selection of a cohort with a lesser degree of other risk factors [39]. In our study, we found no evidence of the obesity paradox, with waist circumference not associated with an increased risk of stroke recurrence.

There are potential limitations to our study. First, patient compliance to prescribed treatment regimens for metabolic syndrome could affect the risk of stroke recurrence, but this could not be evaluated in our study. Second, inclusion of early stroke survivors with only mild-to-moderate stroke severity, and equal distribution of the NIHSS between those with and without metabolic syndrome, limits our study to a subsample of the ischemic stroke population, which may not be representative of the overall ischemic stroke population. Finally, further studies are needed beyond the 1-year follow-up period in order to assess the longer-term prognostic significance of metabolic syndrome, as well as to test the robustness of our data by applying the model to different definitions of metabolic syndrome.

Controversy remains regarding whether the concept of metabolic syndrome is of value in clinical practice [40]. Some think that metabolic syndrome is a defined uniform entity that is relevant to vascular risk factors [41]. Our study suggests that metabolic syndrome may not provide additional information for predicting stroke recurrence beyond consideration of individual factors. However, screening for metabolic syndrome has significant clinical and public health implications for identifying individuals at high risk for vascular disease. It is necessary for metabolic syndrome patients to adopt preventive measures for minimizing recurrent strokes. These measures should include lifestyle modification and pharmacologic treatment directed at ameliorating insulin resistance, hypertension, weight gain, and dyslipidemia.

## Conclusions

Our results suggest that metabolic syndrome may not provide additional information for predicting stroke recurrence beyond the individual factors that compose this syndrome, while high fasting plasma glucose is a significant predictor for stroke recurrence.
